# Framework for Simultaneous Indoor Localization, Mapping, and Human Activity Recognition in Ambient Assisted Living Scenarios

**DOI:** 10.3390/s22093364

**Published:** 2022-04-28

**Authors:** Jesus D. Ceron, Diego M. López, Felix Kluge, Bjoern M. Eskofier

**Affiliations:** 1 Telematics Engineering Research Group, Telematics Department, Universidad Del Cauca (Unicauca), Popayán 190002, Colombia; jesusceron@unicauca.edu.co; 2 Machine Learning and Data Analytics Lab, Computer Science Department, Friedrich-Alexander University, Erlangen-Nürnberg (FAU), 91052 Erlangen, Germany; felix.kluge@fau.de

**Keywords:** simultaneous location and mapping, ambient assisted living, human activity recognition

## Abstract

Indoor localization and human activity recognition are two important sources of information to provide context-based assistance. This information is relevant in ambient assisted living (AAL) scenarios, where older adults usually need supervision and assistance in their daily activities. However, indoor localization and human activity recognition have been mostly considered isolated problems. This work presents and evaluates a framework that takes advantage of the relationship between location and activity to simultaneously perform indoor localization, mapping, and human activity recognition. The proposed framework provides a non-intrusive configuration, which fuses data from an inertial measurement unit (IMU) placed in the person’s shoe, with proximity and human activity-related data from Bluetooth low energy beacons (BLE) deployed in the indoor environment. A variant of the simultaneous location and mapping (SLAM) framework was used to fuse the location and human activity recognition (HAR) data. HAR was performed using data streaming algorithms. The framework was evaluated in a pilot study, using data from 22 people, 11 young people, and 11 older adults (people aged 65 years or older). As a result, seven activities of daily living were recognized with an F1 score of 88%, and the in-door location error was 0.98 ± 0.36 m for the young and 1.02 ± 0.24 m for the older adults. Furthermore, there were no significant differences between the groups, indicating that our proposed method works adequately in broad age ranges.

## 1. Introduction

The continuous advance in the development of ubiquitous devices, characterized by their small size, excellent processing power, and low energy consumption, has allowed progress in many applications fields. Indoor localization (IL) and human activity recognition (HAR) have not been the exception. These two fields are essential for systems that facilitate patient monitoring at home and in systems whose purpose is to offer daily activities assistance, especially in older adults.

Both indoor location and human activity recognition have been widely studied. Many studies have addressed IL and HAR in an isolated way [1]. However, few studies have taken advantage of the IL and HAR relationship. For example, in the context of a house, people usually carry out certain activities in specific places: cook in the kitchen, watch television in the living room or bedroom, wash hands in the bathroom sink, etc. Knowing the exact location of the person might improve the classification of the activity performed. Additionally, information about the action being performed can improve the identification of the individual’s physical location.

Systems used to track the location in indoor environments are usually called indoor positioning systems (IPS). There is a diversity of IPSs that vary according to their technologies [2,3,4]. As stated in [5,6,7], choosing an IPS should be made taking into account seven aspects: the characteristics of the indoor environment, the accepted level of precision, budget limitations, obtrusiveness, system complexity, robustness, and privacy. When choosing the type of IPS to be used in an ambient assisted living (ALL) scenario, these aspects need to be considered. In addition, the indoor localization and recognition of activities of older adults should be carried out to assist them in their daily life tasks. In these types of scenarios, privacy and obtrusiveness are fundamental aspects. Consequently, it is desirable to avoid using IPSs that work with camera-based methods or require the person to wear multiple devices on their body. Under these considerations, we chose to base our proposed framework on the simultaneous indoor localization and mapping (SLAM) framework [8].

SLAM is used to estimate the person’s position in indoor environments. It combines data from proprioceptive sensors such as inertial measurement units (IMU) attached to the body, with exteroceptive sensors, such as cameras, RFID tags, Bluetooth beacons, and laser range sensors configured in the surroundings. A unique feature of SLAM is that it allows to independently create the map through which the person moves. The map is built from the observation of landmarks. These can be objects, devices, or characteristics of the indoor environment [9].

This paper aims to deploy a framework for indoor localization, mapping, and HAR using a non-intrusive configuration (one single IMU placed in the person’s shoe and a set of Bluetooth low energy beacons (BLE) deployed in the indoor environment). The feasibility of the framework is evaluated in a home environment with older adults. Our approach is minimally intrusive and opportunistic since, in addition to tracking the person’s location and type of activity performed, it has the potential to analyze gait parameters in older adults, as shown in our previous work [10]. This approach is very useful for the early detection of gait disorders such as those derived from Parkinson’s disease [11,12].

This study has three main contributions: (1) The collection of a dataset that allows assessing IL and HAR in both adults and older adults; (2) A method for classifying activities based on a data stream learning algorithm [13]; (3) A comprehensive evaluation of a method for simultaneous indoor localization, mapping, and human activity recognition in an AAL scenario with older adults. We believe that the above results contribute to the future development of dynamic systems for monitoring IL and HAR, especially for the AAL.

## 2. Related Work

Few previous works have used one single IMU on the foot to perform simultaneous localization and mapping. Son Gu et al. [14] proposed a method for corner detection based on motion data captured with one IMU attached to one foot. The corridors and corners of the indoor environment were used as landmarks. They evaluated their method in two settings. One was an office environment (20 m × 20 m) and a library (70 m × 70 m). The error in the indoor localization was 1.34 m and 0.8 m, respectively. This error shows that the complexity of the indoor environment is related to the precision obtained. In another study by Robertson et al., RFID tags were used as landmarks [15]. Tags were put on doors and hallways. The reported precision was 2 m in an indoor environment of 20 m × 40 m. In Wi-Fi GraphSLAM [16], Wi-Fi antennas were used as landmarks. The received signal strength indicator (RSSI) from the Wi-Fi antennas deployed in the indoor environment was used to estimate the distance between the person and the antennas. The reported precision was 2.23 m in a 60 m × 10 m environment. In ActionSLAM [17], the landmarks were activities: sitting, standing, opening, or closing doors and windows. The precision achieved was 1.16 m. The main disadvantage of this method is the need to use two additional IMUs to the one worn on the foot: one on the waist to detect when the person sits or stands, and another in the hand to detect opening or closing doors or windows. BLE beacons were used as landmarks by Zuo et al. [18]. The method was evaluated in an area of 90 m × 37 m. Its accuracy was 3.25 m using 48 beacons and 4.69 m using 24 beacons. In [19], a pilot test of our authorship, we used BLE beacons as landmarks in a simulated indoor environment of 16 m × 7 m. The precision obtained was 1.05 m ± 0.44 m.

Recognizing human activity, especially in AAL scenarios, involves dealing with data that changes or evolves over time. The way in which an older adult performs activities of daily living can change over time due to the natural aging process or a specific condition, such as Parkinson’s. Traditional supervised learning has been extensively used to obtain classification models for HAR. However, this type of approach does not have the capacity to easily update the generated classification models. Data stream algorithms have the ability to update their classification model with each sample they classify [13]. Therefore, they can deal with dynamic systems, such as HAR in AAL. Khannouz et al. [20] compared the performance of five data stream algorithms using two real [21,22] and three synthetic HAR datasets. The real datasets used by Khannouz et al. do not contain activities of daily living. They only include data from IMUs. As a result, the Mondrian Tree and Naive Bayes algorithms obtained the best F1 measures. The data stream algorithms of the StreamDM-C ++ [23] and OrpailleCC [24] apps were used in that work.

## 3. Materials and Methods

The framework was constructed using simultaneous indoor localization, mapping, and human activity recognition, previously proposed by the authors [19]. This method is the first to use a combination of BLE beacons and HAR as landmarks. In this paper, we propose an improved HAR method using a data stream algorithm. The data stream paradigm was created in response to the need to deal with a continuous stream of data sampled in real-time [13]. The most important characteristic of algorithms written for data streams is updating their model by inspecting each training example. This means that the model can be updated without splitting a static dataset into training and test sets. In addition, as they inspect each training example once, there is no risk of memory overflow, and usually, the processor load is very low. The characteristics of this type of algorithm are appropriate for HAR because: (1) they are capable of working in real-time, i.e., processing and classifying the examples that arrive one by one; (2) they optimize the data processing, reducing memory use. Therefore, they can efficiently run on mobile and wearable devices with reduced memory and battery capacity; (3) Unlike traditional classification algorithms, data stream algorithms can maintain up-to-date classification models. This feature is particularly important in AAL settings, in which the way older adults perform activities of daily living may change over time.

### 3.1. Dataset

**Figure 1:**
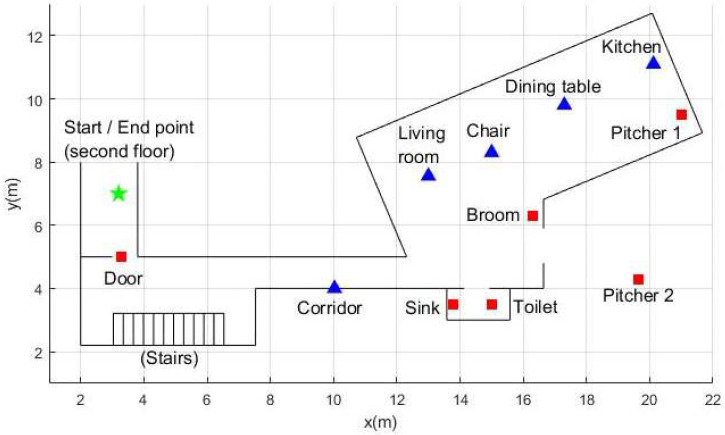
Map of the house. Top view of the house where the dataset was collected. The green star indicates the start and end coordinates of data collection. The blue triangles represent stationary beacons, and the red squares are active or mobile beacons.

The data used in this research were collected in a house in Popayán, Colombia (Figure 1). In total, 22 people performed 9 different activities, as described in Table 1. Overall, 11 people were older than 60 years (older adults’ group) with an average age of 70.72. The average age of the remaining 11 people was 41.81 (control group). Three types of data were collected: data of the movement of the person’s foot, the person’s proximity to Bluetooth beacons deployed in the house (Figure 1), and the motion of the beacons attached to daily objects. The average duration of data collection for each participant was 7.4 min. The ethics committee approved the collection protocol of this dataset of the University of Cauca. Before data collection, the participants signed informed consent. All participants are able to perform activities of daily living based on their answers to the Katz Index (measuring independence in activities of daily living).

#### 3.1.1. Foot Movement Data

**Figure 2:**
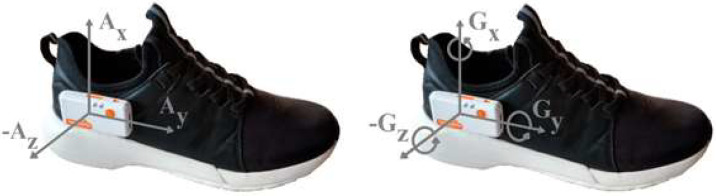
Axis alignment of the IMU attached to the right shoe of each participant.

The person’s foot movement data collected was their acceleration and angular velocity. For this, an IMU (Shimmer sensing, Dublin, Ireland) was attached to each participant’s lateral part of the right shoe (Figure 2). The IMU was previously calibrated, and a sensitivity of ±16 g was used for acceleration and ±2000°/s for angular velocity. The sampling frequency used was 204.8 Hz.

#### 3.1.2. Proximity Data

Then, 10 BLE beacons (Estimote proximity beacons, Estimote Inc., San Francisco, CA, USA, https://estimote.com, accessed on 10 March 2022) were used (Figure 1). A mobile application was developed to run in the background on a smartphone that each participant carried in each session to receive the data streamed by the beacons. The beacons were configured to transmit 10 packets per second. Each packet contains the received signal strength indicator (RSSI). This indicator relates the distance between the smartphone and a given beacon through the equation known as the log-distance path loss model:

(1) $$RSSI=10n\;\log_{10}\left(\frac{d}{d_{1m}}\right)+RSSI_{1m}$$

The transmission power used in all beacons was −20 dBm. According to the manufacturer, this power coverage is achieved in a radius of 3.5 m. After experimenting with the beacons, we conclude that the RSSI at a distance of one meter is approximate −80 dBm. Therefore, $d_{1m}=1\;m$ and, $RSSI_{1m}=-80$. The n term depends on the signal transmission medium. To obtain a consistent relationship between RSSI and distance in Equation (1), n = 0.6 was used. The variable d is the distance between a given beacon and the smartphone that the person carries.

#### 3.1.3. Motion Data from Beacons Attached to Objects (Active and Mobile Beacons)

In addition to RSSI, the packets sent by the beacons contain three-dimensional acceleration signals of the beacon. This functionality allows the movement of the beacon to be directly related to the use of the object to which it is attached.

**Figure 3:**
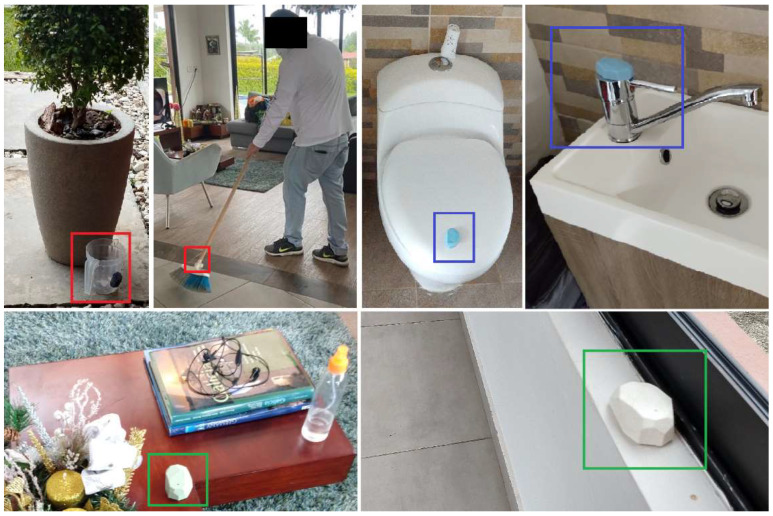
Beacons deployed. The two mobile beacons are in red squares (in jug and broom). Two of the six active beacons are in blue squares (toilet and bathroom sink). Two of five stationary beacons in green squares (living room and corridor).

Depending on its purpose, a beacon can be categorized as stationary, active, or mobile (Figure 3):

1. Stationary beacons: Beacons intended to be stationary in a defined place, for example, on the table or in the kitchen. Their movement is not of interest. They are only used to estimate proximity.
2. Active beacons: An active beacon is attached to an item related to a daily life activity, for example, the toilet and the bathroom sink. In this way, the movement of an active beacon can indicate that the person is carrying out the activity related to that beacon. For example, if it is detected that the beacon placed on the bathroom sink is moving, this could indicate that the person is washing their hands.
3. Mobile beacons: Beacons that are also attached to an object of daily use, whose movement indicates an activity. Still, unlike active beacons, the position of mobile beacons can change drastically since they are attached to elements, such as the broom and a jug.

The deployment of the beacons, especially the stationary beacons, was carried out following the recommendations of Castillo-Cara et al. [25]. They conclude that the transmission power and the number of beacons are the main parameters to perform indoor localization. Therefore, they recommend dividing the area into sectors. Each sector must be covered by a beacon signal. The sectors must be far enough apart to avoid overlapping the Bluetooth signal. They also recommend using a transmission power that is not too high to avoid signal multipath problems.

### 3.2. Proposed Framework

**Figure 4:**
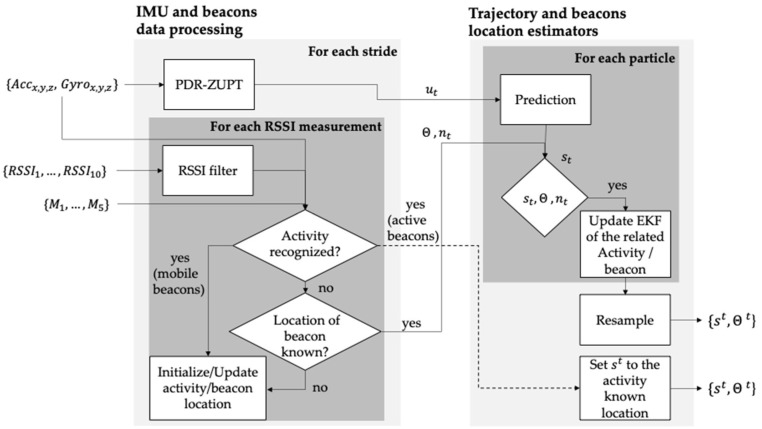
Proposed framework. The left block receives IMU and beacons data, executes a pedestrian dead-reckoning algorithm, initializes the beacon’s location, and recognizes the human activities. The block on the left implements the three phases of a particle filter algorithm: prediction, update, and resample. As a result, the person and beacon’s indoor location are obtained.

The framework for indoor localization, mapping, and human activity recognition improves our previously presented method [19]. This new approach adds a module for human activity recognition that uses the data stream algorithm. The main components of the proposed framework are described in Figure 4.

#### 3.2.1. IMU and Beacons Data Processing Block

In the “IMU and beacons data processing” block (Figure 4), the data of the IMU and the beacons are processed, the initial estimation of the position of the beacons is made, and the recognition of the activities begins. With the results obtained in this block, the “Trajectory and beacons location estimators” block is in charge of updating the map of the house and the position of the person in it.

##### PDR-ZUPT

The acceleration data from the IMU feeds an algorithm that implements the pedestrian dead reckoning technique (PDR) with zero velocity update (ZUPT). PDR ZUPT has been proven to be an excellent strategy to reduce drift intrinsically caused by the use of accelerometers and gyroscopes [26]. In addition, the PDR ZUPT module generates an estimate of the length and orientation of each step. With these data, an estimate of the person’s current position in the Cartesian plane is obtained. This estimate is called the motion measurement $u_{t}$. Finally, that is sent to the prediction module of the “Trajectory and beacons location estimators.”

##### RSSI Filter

Like all RF signals, the Bluetooth signal is susceptible to multipath propagation and fading [27]. Therefore, it is necessary to filter the RSSI data before it can be used to obtain an adequate estimate of the proximity between the person and the beacons. The filter used for this purpose is a one-dimensional Kalman filter [19].

##### Human Activity Recognition Module

The SLAM method mitigates the error derived from calculating the motion measurement $u_{t}$ obtained with the PDR-ZUPT method by observing landmarks [28]. Furthermore, the set of all landmarks detected at time t $\theta =\left\{\theta_{1},\;\theta_{2},\;\ldots ,\theta_{t},\right\}$ represent the map through which the person is moving. In this work, the 10 beacons described above were used as landmarks. There is a specificality with active and mobile beacons since their movement is related to a specific activity. In [19], we established a simple rule to relate the motion of beacons to an activity: if it is detected that a beacon is being moved, it was presumed that the person was using that beacon and, consequently, was carrying out the activity related to it. However, this assumption can cause false positives in multi-inhabited environments, since it might be that the cause of the movement of a beacon is not the person who is using the system. For that reason, there is a need to classify human activity more rigorously, taking as input the data from the beacons and the movement data collected with the IMU.

Because the specific problem of recognizing human activity involves dealing with data that comes from a dynamic system, that is, that changes or evolves over time, the technique to perform the recognition of human activity should be able to deal with this problem. That is why data stream algorithms were chosen.

The data collected from the IMU and the beacons were synchronized using scripts written in Python. Four of the activities to be classified are directly related to active and mobile beacons (Table 2). The labeling of each dataset sample with its corresponding activity was carried out manually with an application developed in MATLAB that allows synchronizing the previously synchronized data with the video of its collection.

After the RSSI data has been filtered $\left\{RSSI_{1},\;RSSI_{2},\;\ldots ,RSSI_{10},\right\}$, it is used together with the motion data of the beacons $\left\{M_{1},\;M_{2},\;M_{3},M_{4}\right\}$ and the acceleration and angular velocity data {Acc, Gyro} to perform the feature extraction process. This process consisted of taking 5-s windows of data (1024 samples) and extracting features from them. The features initially extracted were 40, which included:

1. The maximum and minimum value for each axis of the accelerometer and gyroscope (n = 12);
2. Mean, median, and standard deviation of the acceleration and angular velocity, respectively, for each accelerometer axis (n = 18);
3. The mean of the square root of the sum of the values of each axis squared (mean energy of the accelerometer and gyroscope signal, n = 2);
4. The ID of the four closest beacons detected (n = 4);
5. State of movement of beacons: binary variables that indicate if the active and mobile beacons are in movement (n = 4).

The windows of 5-s of data were used according to the results obtained in [29]. The correlation-based feature subset selection algorithm was used to select the most relevant features. This algorithm detects the subset of highly correlated features with the class while having low intercorrelation [30]. As a result, nine features were selected by the algorithm using ten-fold cross-validation in all the datasets:

1. Maximum acceleration value of the x-axis;
2. Median acceleration of the z-axis;
3. Median angular velocity of the z-axis;
4. Gyroscope energy;
5. Movement status of the active and mobile beacons (2 active and 2 mobile beacons);
6. The ID of the closest beacon.

These nine features were used to train and evaluate three data stream classification algorithms: Naive Bayes, Hoeffding tree, and K-nearest neighbor (KNN). The algorithms Hoeffding tree and KNN are adaptations of the original algorithms to fulfill the conditions of the data stream classification. On the other hand, the original Naive Bayes algorithm does not need to be adapted for data stream classification because it is trained incrementally based on Bayes’ theorem, and its memory requirements are low.

Summary of the functioning of the human activity recognition module:

For each $RSSI_{x}$ reading (coming from beacon *x*) greater than −85 dbm, it is checked if beacon x is in motion or if the reading belongs to a 5-s window classified as one of the activities 4, 5, 6, or 7 (Table 2). If so:

1. If the RSSI comes from a mobile beacon in motion, its position is updated in the “update activity/beacon location” module. If the RSSI comes from an active beacon in motion, the person’s position at time *t* $\left(S_{t}\right)$ is set equal to the known location of the beacon;
2. If the RSSI comes from a stationary beacon, it is checked whether the location of beacon x is known.
  - If so, the set of landmarks at time t $\theta =\left\{\theta_{1},\;\theta_{2},\;\ldots ,\theta_{t},\right\}$ is sent to the block “trajectory and beacons location estimators” together with the array of IDs of the beacons detected at the time $\left(n_{t}\right)$;
  - If not, its position is updated in the “update activity/beacon location” module.

##### Update Beacon/Activity Location

When starting the execution of the proposed framework, the location of all beacons is unknown. In consequence, their location must be initialized. We developed a method based on the particle filter for this purpose [31]. For stationary and active beacons, the method estimates the distance between the beacon and the person (smartphone) and assumes that the error in this estimate is 10%. For example, if the estimated distance between the person and the beacon is 2 m, the beacon should be within a circle with a radius between 1.8 m and 2.2 m. The 10% value was obtained by recording the RSSI of a beacon with a smartphone at different distances from 0 m to 3.5 m every 0.5 m. The distance from the smartphone to the beacon was calculated using Equation (1). The maximum distance error (difference between the actual distance and the calculated distance) obtained was close to 10% (~35 cm). This process was executed twice using two different beacons. Subsequently, 2000 particles are created uniformly distributed on that disk. Each particle represents a possible location of the beacon. Each time a new RSSI reading is received from the beacon, the particle filter updates until it finally converges on a point on the map. How the position of mobile beacons is initialized and updated is similar to that described for stationary and active beacons. It only differs that instead of creating a disk, a sphere is created. Inside it, the particles are uniformly distributed.

#### 3.2.2. Trajectory and Beacon’s Location Estimators

At the beginning of data capture, and each time the current position of the person $s_{t}$ is updated when an activity is recognized, a particle filter is responsible for creating 600 particles, each of them representing a possible trajectory $S_{t}$ of the person. With each motion measurement $u_{t}$ generated when the PDR-ZUPT module detects a step, the particle filter prediction phase estimates the new state of each particle. The current state of each $s_{t}$ particle represents a possible x–y position of the person. The possible trajectories followed by the person are given by the set of previous states of each $S_{t}$ particle. Each particle has an associated weight *w* that represents the probability of describing the person’s true trajectory.

**Figure 5:**
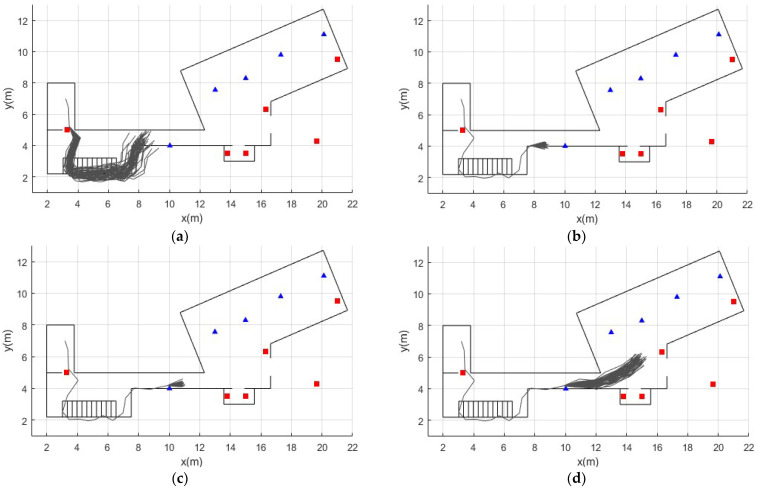
Example of obtaining a trajectory with the particle filter. Blue triangles represent stationary beacons. Red squares represent active and mobile beacons. (**a**) The person walks from the second floor into the corridor. In the prediction phase, the filter estimates the possible trajectories, denoted by dark gray lines. (**b**,**c**) the person enters the range of a stationary beacon placed in the corridor. The particles are updated with the RSSI data from that beacon, and the resampling process is executed. The filter converges in a single trajectory (**d**). The person continues on his way to the kitchen. The process is repeated.

The location of each beacon is calculated independently with extended Kalman filters (EKF). Since beacons only provide information about their proximity but not orientation, the EKF had to be adapted based on the range-only SLAM method proposed by Menegatti [31]. Every time an RSSI reading is received from a beacon whose location is known, the distance between the beacon and the person’s position given by the current state of each particle is calculated. With these data, the EKF updates the beacon’s position and the weight w of each particle. Finally, the resampling process eliminates particles with very low weights and replaces them with those with higher weights. This process is performed by the systematic resampling method [32]. In conclusion, the framework is able to locate the beacons from scratch based on the information from the PDR-ZUPT module and the RSSI measurements of the beacons. Therefore, the information of the position of the beacons and the location of the person is complemented and updated as the person moves in the indoor environment. An example of how the particle filter is responsible for estimating the person’s trajectory can be seen in Figure 5.

### 3.3. Evaluation

The evaluation process for the human activity recognition module and indoor localization is detailed below. In addition, all the software developed in Python and MATLAB for the acquisition and processing of data and the dataset used for the evaluation (presented in Section 3.1) is publicly available.

#### 3.3.1. Human Activity Recognition

The software “Massive Online Analyzes” (MOA) [13] was used for the evaluation of the human activity recognition module. This software, written in Java, contains a pool of data stream classification algorithms and offers an API that provides many features, including training and evaluation, obtaining evaluation metrics, and exporting the created models for later use.

The evaluated algorithms were used with their default parameters. For example, the number of activities to be recognized was 7. Consequently, K was set to 7 in KNN.

The evaluation method used is called interleaved Test-Then-Train. In this, each example is used to evaluate the model before it is used to train it. In this way, the model is continuously assessed with unseen examples, ensuring the model’s generalization. In addition, this method enables obtaining updated statistics with each example that the algorithm processes, thus drawing its learning curve.

We used the F1 score to evaluate the performance of the classifiers. This metric is a measure that gives equal importance to precision and recall and is frequently used to assess the performance of models generated from unbalanced datasets, such as the one collected in this study:

(2) $$F1=2\frac{precision*recall}{precision+recall}$$

In total, 3716 examples were extracted from the data of the 22 participants. On average, there were 169 examples per participant. The evaluation process was similar to the leave-one-subject-out method. As the data stream classification algorithm is trained example by example, an arff file was created in which the examples of the participant were placed at the end of the file, after the examples of the other 21 participants. In addition, the examples of the 21 participants were randomized in each file to obtain results that show the variance of the F1 measure between participants and hinder the algorithms’ learning process.

#### 3.3.2. Indoor Trajectory Reconstruction

The complete trajectory followed by each participant during data collection can be divided into six sub trajectories:

1. From the main entrance to the kitchen (where a drink is served with the jug);
2. From the kitchen to the sink (person washes his hands in the sink);
3. From the sink to the garden (water the plant with a jug);
4. From the garden to the living room (sweep the living room with the broom);
5. From the living room to the bathroom (use or pretend to use the toilet);
6. From the bathroom to the main entrance.

Except for the initial and final activity, the rest of the sub-trajectories begin and finish when an activity is detected. Thus, the start and end coordinates of data collection are the same. In addition, the exact starting and ending coordinates of each sub-trajectory are known. Therefore, it is possible to calculate the error of the estimated trajectory by calculating the Euclidean distance between the coordinates of the last step of the sub-trajectory estimated and the true coordinates where the activity starts.

It is interesting to demonstrate that the proposed framework can work appropriately for adults and older adults. Therefore, a Student *t*-test was performed to compare the means of the location errors between the two groups. The null hypothesis states that there is no significant difference between the means of the indoor localization errors of the older adults and control groups. A 95% confidence interval was used.

## 4. Results

### 4.1. Human Activity Recognition

To evaluate the three data stream classification algorithms’ overall performance, Table 3 shows the precision, F1 score, and processing time to process the 3716 examples. The data in each column correspond to the average of all participants.

The main evaluation metric used to evaluate HAR was the F1 measure. Table 4 shows in detail the average value of this metric for each sub-trajectory.

**Figure 6:**
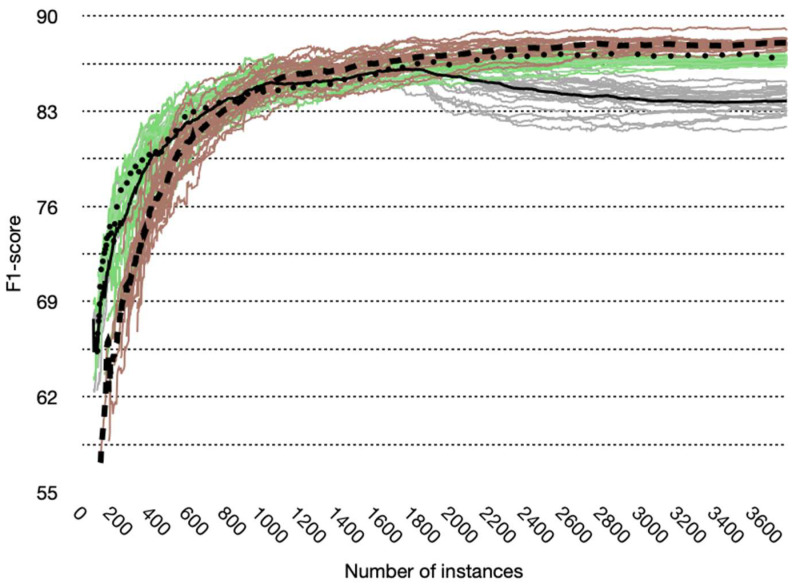
Learning curves for all participants and their average. NB: green lines, average on the dotted line. HT: gray lines, average on the solid line. KNN: red lines, average in broken line.

The interleaved Test-Then-Train evaluation method allows obtaining the learning curve of the data stream algorithms (Figure 6). Each example that the algorithm receives evaluates the model created so far and then uses the same example to train and update the model.

In addition, a *t*-test was carried out to test for any significant difference in the F1 measure of the older adults and control group for each activity (Table 5). There was a significant difference between the two groups only in the case of the recognition of the walking activity.

### 4.2. Indoor Localization Error

The generation of particles in the prediction phase is a random process. This means that the proposed method must be executed several times to have a reasonable estimate of the indoor location error for each participant. Therefore, the proposed method was run 10 times for each participant. Table 6 and Table 7 show the average location error in each sub-trajectory for the older adults and control group, respectively.

The mean error in the location of the older adults’ group was 1.023 m, and that of the control group was 0.986 m.

**Figure 7:**
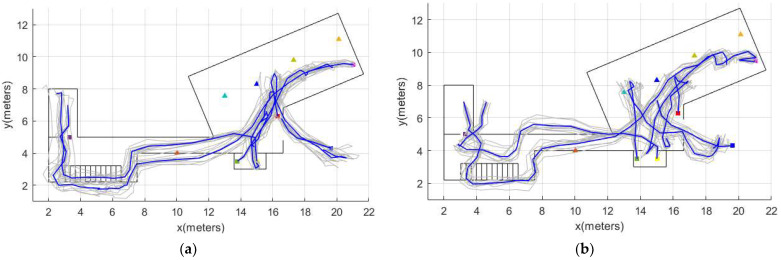
Example of location tracking. (**a**) Participant 9 of the older adults group. (**b**) Participant 2 of the older adults group.

Figure 7 shows an example of the location tracking for two participants. The location tracking results for all the 22 participants can be shown in Appendix A.

There are no significant differences in the location error between older adults and the control group (Table 8).

## 5. Discussion

The three main contributions of the paper are discussed as follows: (1) The collection of a dataset that allows assessing IL and HAR in both adults and older adults; (2) A method for classifying activities based on a data stream learning algorithm; (3) A comprehensive evaluation of a method for simultaneous indoor localization, mapping, and human activity recognition for AAL scenarios.

### 5.1. Dataset

No single dataset was found with data that allow the evaluation of the proposed framework. Therefore, the collected dataset constitutes a fundamental part of our research, and it is publicly available to other authors. It included synchronized data from the IMU that each participant carried on their right foot and the BLE beacons deployed in the indoor environment. In addition, each example was labeled with one of the seven activities that the participants performed (Table 2). The collected dataset includes data from 22 people, of which 11 are older than 60 years. This allowed us to compare the framework’s performance concerning HAR and IL with adults and older adults. Finally, the dataset was collected in Colombia, allowing the training of local models in low-and middle-income settings and it has been widely recommended in the literature [33,34].

### 5.2. Human Activity Recognition

In our work, HAR was carried out using three data stream algorithms: K-Nearest Neighbor (KNN), Naive Bayes (NB), and Hoeffding Tree (HT). The percentage of the examples correctly classified using these three algorithms was high. KNN and NB almost reached 90%, and HT reached 87.56%. However, the results of the F1 measure are a little lower, but the order of algorithms concerning their performance is preserved: the best F1 is KNN (88.01%), followed by NB (86.78%), and, finally, HT (83.76%) (Table 3). In Khannouz’s work, the level of F1 was much lower, although a direct comparison of the performance of the algorithms cannot be made because the datasets used are different. For example, their real datasets do not contain data from BLE beacons, and the activities carried out in them are different.

The activity of going up or down the stairs produced a low F1 in all cases. On a closer inspection, the reason for this is that algorithms classified many examples labeled as “climbing/descending stairs” as “walking.” Consequently, the number of false positives affects the F1 measure of the “walking” activity. This phenomenon seems to affect more to the older adult group. Consequently, there was a significant difference in recognition of the walking activity between older adults and control groups (Table 5). There are ways to handle the low F1 obtained for the “Climbing/descending stairs” activity. The simplest solution is to merge the activities “walking” and “Climbing / descending stairs.” In that case, when looking at the results in Table 4, it is foreseeable that KNN and NB would have a very similar F1. Another option would be to look for features that help classify that activity and include them in the learning phase. It would also be possible to place two stationary beacons on both sides of the stairs, which in addition to helping to refine the location, would help to classify the activity in question. Finally, including the data from a barometer is a viable option. Some IMUs have a built-in barometer, and it has already been proven as a valuable sensor for measuring changes in height in indoor environments [35,36].

Although the learning curves for the KNN and NB algorithms maintain an upward trend after 1800 examples, the learning curve for the HT algorithm has a downward behavior that stabilizes approximately at example number 3000. Consequently, the algorithms KNN and NB are the candidates to be chosen as the algorithm to include in our framework. Furthermore, the learning curve of the KNN algorithm is lower than the curves of the NB and HT algorithms up to approximately 1000 examples. This behavior is expected since KNN must base its classification on the most similar examples previously classified. Still, since it is a data stream algorithm, it does not save all the history of the examples from performing the comparison. Instead, it only stores some [13].

The evaluation of a data stream algorithm includes calculating the time it takes to process each example. The processing time of the three algorithms has a linear behavior through time. Therefore, the time it takes for the algorithm to process an example corresponds to the division of the total time it took to process the 3716 examples by the total number of examples. The nearest neighbor search performed in KNN and the need to store a set of examples to compare the similarity with unknown examples is costly computational tasks compared to the tasks required in NB and HT. However, KNN processes each sample in 12.9 ms, and the proposed framework needs to classify an example every 5 s. In addition, the classification model obtained with KNN could be quickly executed on a device with a very low memory capacity since it only uses 0.69 kB.

Another important result is that the *t*-test showed no significant difference between the means of the F1 measure of the older adults and the control group (Table 8). As a result, it can be stated that the KNN data stream algorithm can classify the activities of both groups satisfactorily.

### 5.3. Simultaneous Indoor Localization, Mapping, and Human Activity Recognition

The total average localization error was 1.023 m for the older adults group participants and 0.986 m for the control group. These results are similar to those obtained in our previous works [10,19] and the reported in the related work section (Table 9).

It is not appropriate to make a direct comparison between the studies in Table 9 since each of them was evaluated under different conditions, that is, with different devices, different indoor environments, and different number of beacons. However, it is important to note that the IL precision of the proposed framework is better than the reported in the other works. The analysis made by [18] is important since they improved the precision by increasing the number of beacons from 24 to 48. The novelty of our study is the comparison of the results between the groups. To the best of our knowledge, this is the first study comparing the precision of IL in adults and older adults.

In [10], we demonstrated that the proposed PDR-ZUPT module was capable of reconstructing trajectories that involved walking, jogging, and running. That result was an indication that the proposed final framework would be able to locate people while they are walking at different speeds. This is important because the gait of older adults is usually slower than in adults. As a result of the evaluation, there was no significant difference in the location error between older adults and control groups. This result, along with the results obtained in the HAR section, indicates that the proposed framework to perform simultaneous indoor localization, mapping, and human activity recognition works adequately with older adults, and, therefore, is suitable to be implemented in ambient assisted living scenarios. It is important to note that this result implies that the inclusion of the data stream classifier for HAR was successful since the proposed framework follows the location based on the results of the HAR module.

Going deeper into the comparison of the results of the two groups, the largest average error occurs for the sub-trajectory between going from the kitchen to the bathroom sink. That trajectory implied that the participant served something to drink in the kitchen, walked to the dining room, sat down and ate something, stood up, and went to wash their hands. It is known that sitting activities are likely to cause loss of heading, even more, when the person moves her feet when is seated. One way to deal with heading drift is to deploy as many beacons as possible and position them to have a line of sight with the Bluetooth signal receiver device as much as possible. This design consideration must be considered if the receiving device is the same IMU put in the person’s shoe. In that case, it would be preferable for the stationary beacons to be kept level with the ground.

**Figure A1:**
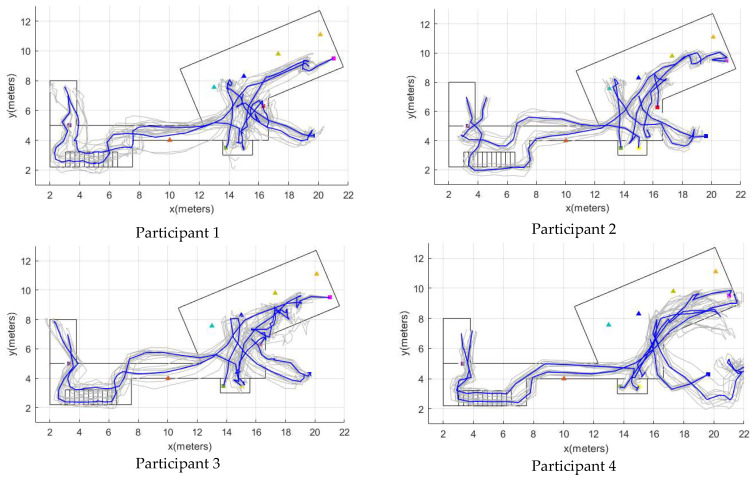

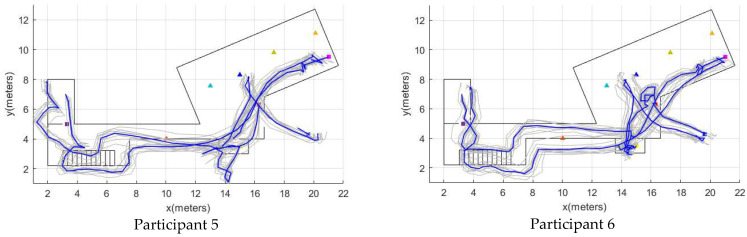
Indoor localization results (Older adults’ group, participants 1–6).

There is a drift in reconstructing the final sub-trajectory of participants 1 and 5 of the control group (Figure A3) and participant 2 and 5 of the older adults’ group (Figure A1). That drift results in a large error in the location of said sub-trajectory for the participants mentioned above (Table 6 and Table 7, respectively). The activity consists of going from the bathroom to the main entrance on the second floor. The loss of heading occurs from the moment the sub-trajectory begins, just after using the toilet. As in the previous case, having more beacons to correct the orientation would be very useful. As proposed in Section 5.2, placing a beacon at each end of the stairs would improve heading drift and classify the “climbing / descending stairs” activity.

**Figure A2:**
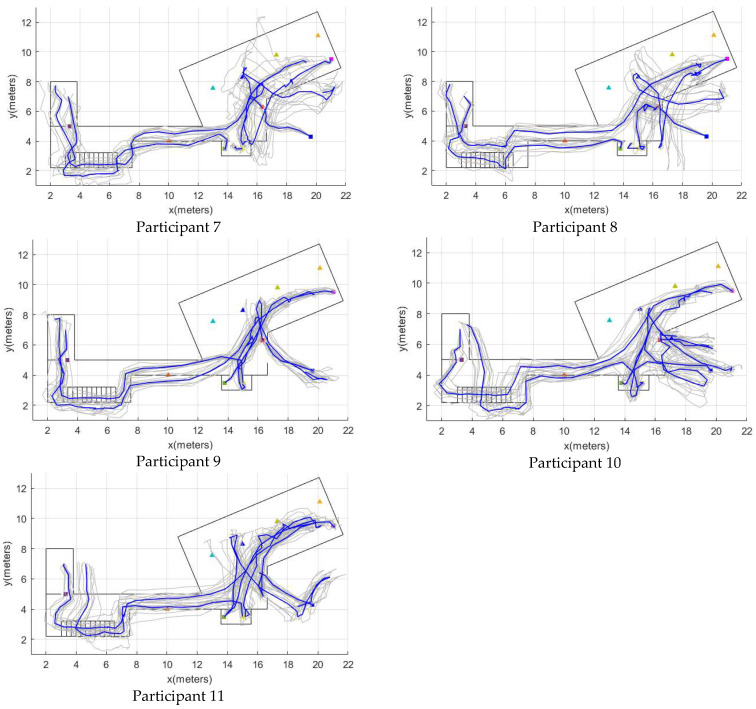
Indoor localization results (Older adults’ group, participants 7–11).

**Figure A3:**
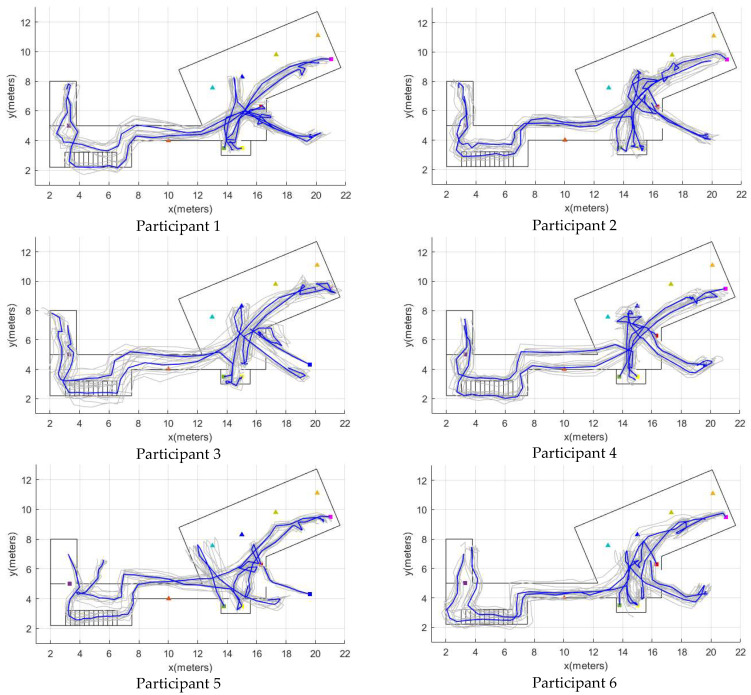
Indoor localization results (control group, participants 1–6).

**Figure A4:**
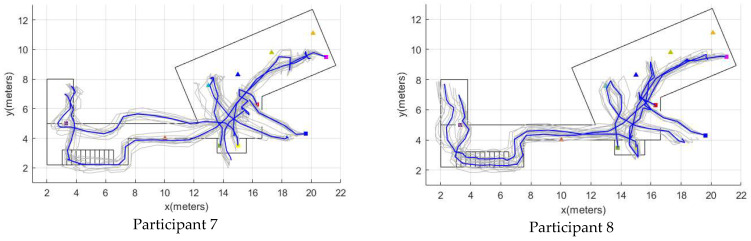

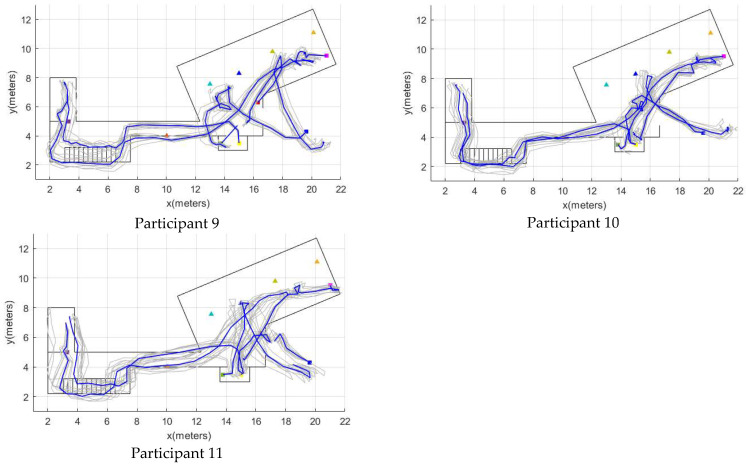
Indoor localization results (control group, participants 7–11).

The longest sub-path (around 20 m in length) is to go from the starting point to the kitchen. Although this activity involves going downstairs, the indoor localization on the stairs worked very well for both groups (Figure A1, Figure A2, Figure A3 and Figure A4). However, the average location error for this sub-trajectory was 1.95 m for the control group and 1.287 m for the older adults group. Then, the cause of the error is in the correction of the position carried out by the particle filter. As in the case of heading loss, they have more beacons, especially stationary ones, that are at the same level as the receiving device of their signals, which would be the way to improve localization.

## 6. Conclusions

We have presented a framework that takes advantage of the relationship between location and activity to simultaneously and non-invasively perform indoor localization, mapping, and human activity recognition. The framework fuses data from an IMU placed in the person’s shoe with proximity and human activity-related data from BLE beacons. The feasibility of the framework was demonstrated in a smart home environment with adults and older adults performing simultaneous location, mapping, and recognition of daily life activities.

The results show that SLAM allows a dynamic indoor localization. The recognition of activities aided with the interaction with Bluetooth beacons constitutes an excellent alternative to landmarks in SLAM. This allows mapping the area by which the person moves and refining the localization of the person in it. Although previous works have used BLE beacons for indoor localization, to the best of our knowledge, this is the first one that uses the movement of beacons as an input variable for classifying activities that are later used as landmarks in the SLAM framework. HAR was carried out with a KNN data stream algorithm, which can be executed on any wearable because its little memory consumption. In a future work, to have the HAR classification model up to date, an automatic process to label the learning examples could be performed based on the movement of the beacons and their proximity to them.

The proposed framework is the backbone for future projects to develop flexible systems for monitoring IL and HAR based on two non-intrusive components: an IMU and Bluetooth beacons. The IMU in the shoe and BLE beacons deployed in the house are the necessary devices for the operation of the proposed framework. The smartphone was required since the IMU used does not have the functionality to receive Bluetooth signals. However, it is possible to adapt or develop an IMU capable of avoiding using the Smartphone. The use of the IMU at the foot, in addition to being slightly intrusive, is opportunistic because it facilitates the detection of gait parameters in older adults. It is beneficial for detecting gait disorders, such as those derived from Parkinson’s disease [37]. As future work, the development of a smart insole that contains one accelerometer and gyroscope and the capacity of receiving the BLE signal from the beacons can be beneficial to increase the use comfortableness. Other interesting future work consists of evaluating the framework in different indoor environments to demonstrate its dynamism. In addition, it is proposed to develop a software solution for the local or remote processing of the framework that allows identifying a specific user and visualizing their location and activities.

## Figures and Tables

**Table 1:** Activities carried out by participants during data collection.

Activity	Description
Enter the house	The session begins on the second floor of the house.
Serve something to eat	Go down the stairs, go to the kitchen and use a jug to serve something to drink
Eat at the dining table	Take what was served to the dining room and drink it. Food is also available at the dining table if desired.
Wash hands	Go to the bathroom and wash hands in the bathroom sink
Watch TV	Go to the living room, sit on the couch, and watch TV for at least two minutes
Water a plant	Go to the garden, take the jug, and water a plant
Sweep the floor	Take the broom and sweep for at least one minute
Use the toilet	Go to the bathroom and use it or pretend to use it (lift the toilet lid, wait a few seconds, and lower it)
Leave the house	Head towards the same starting point on the second floor.

**Table 2:** Human activities recognized by the HAR module.

	Activity	Beacon Related	Type of Beacon
1	Walking	NA	NA
2	Climbing/descending stairs	NA	NA
3	Being still	NA	NA
4	Using jug	Beacon in pitcher	Mobile
5	Sweeping	Beacon in broom	Mobile
6	Using bathroom sink	Beacon in bathroom sink	Active
7	Using toilet	Beacon in toilet	Active

**Table 3:** General performance of algorithms.

	F1 Score (Percent)	Evaluation Time (CPU Seconds)	Classifications Correct (Percent)
KNN	88.01	45.68	89.92
Naive Bayes	86.78	0.51	89.70
Hoeffding tree	83.76	0.73	87.56

**Table 4:** Performance of the algorithms in the classification of each activity.

	Walking	Climbing/Descending Stairs	Using Pitcher	Being Still	Using Bathroom Sink	Sweeping	Using Toilet
KNN	82.24	49.87	99.41	94.60	91.57	99.50	96.84
Naive Bayes	82.21	31.29	99.58	95.13	91.53	99.61	98.27
Hoeffding tree	79.63	29.71	97.28	93.82	85.06	98.27	92.52

**Table 5:** Student’s *t*-test result. Comparison of F1 measure between older adults and control group.

	F1 Mean (Control Group)	F1 Mean (Older Adults Group)	Mean Difference	Std. Error Difference	*p*-Value
Walking	82.41	82.06	−0.35	0.17	0.05
Climbing/descending stairs	49.82	49.91	0.09	0.88	0.91
Using pitcher	99.42	99.39	−0.02	0.03	0.49
Being still	94.56	94.63	0.07	0.09	0.45
Using bathroom sink	91.48	91.65	0.16	0.19	0.39
Sweeping	99.50	99.49	−0.01	0.03	0.71
Using toilet	96.71	96.95	0.23	0.15	0.14
All activities	88.02	88.00	−0,01	0.13	0.90

**Table 6:** Average error (in meters) of the location for each sub-trajectory (older adults’ group).

Participant	Door to Kitchen	Kitchen to the Bathroom Sink	Bathroom Sink to Plant	Plant to Room	Broom to Toilet	Toilet to Door	Total Average Error
1	1.928	1.504	0.496	1.568	0.447	0.568	1.085
2	1.752	1.303	1.220	1.499	1.376	1.682	1.472
3	1.981	1.497	0.403	0.747	0.446	0.758	0.972
4	0.453	1.024	1.994	0.620	1.059	0.990	1.023
5	1.209	0.938	0.631	3.058	0.301	1.181	1.220
6	0.633	1.135	0.724	0.224	0.686	0.785	0.698
7	1.679	3.055	0.319	0.535	0.681	0.834	1.184
8	2.095	3.279	0.299	0.151	0.475	0.881	1.197
9	1.248	1.367	0.559	0.284	0.151	0.598	0.701
10	1.297	0.761	1.385	0.901	0.391	0.683	0.903
11	1.069	0.174	1.015	0.224	0.591	1.697	0.795
Average	1.395	1.458	0.822	0.892	0.600	0.969	1.023

**Table 7:** Average error (in meters) of the location for each sub-trajectory (Control group).

Participant	Door to Kitchen	Kitchen to the Bathroom Sink	Bathroom Sink to Plant	Plant to Room	Broom to Toilet	Toilet to Door	Total Average Error
1	0.912	0.872	0.307	0.613	0.181	0.221	0.518
2	1.061	0.367	0.347	0.477	0.233	0.360	0.474
3	0.995	1.510	1.513	0.855	0.975	0.945	1.132
4	1.133	0.907	0.643	0.442	0.288	0.284	0.616
5	0.710	2.137	2.916	0.234	0.198	2.767	1.494
6	1.413	1.919	0.545	0.464	0.257	0.887	0.914
7	1.046	1.143	1.367	0.607	1.075	0.948	1.031
8	1.238	1.254	1.590	1.133	0.268	0.798	1.047
9	1.731	2.427	2.154	1.359	1.072	0.241	1.498
10	0.957	1.322	1.516	0.166	0.345	0.415	0.787
11	2.955	1.718	1.148	1.107	0.227	0.867	1.337
Average	1.287	1.416	1.277	0.678	0.465	0.794	0.986

**Table 8:** Student’s *t*-test result. Comparison of errors between older adults and control group.

	Error Mean (Control Group)	Error Mean (Older Adults Group)	Mean Difference	Std. Error Difference	*p*-Value
Main door to the kitchen	1.28	1.28	−0.00	0.29	0.90
Kitchen to bathroom sink	1.41	1.41	0.18	0.34	0.60
Bathroom sink to plant	1.27	1.27	−0.49	0.30	0.11
Plant to broom	0.67	0.67	0.21	0.29	0.47
Broom to toilet	0.46	0.46	0.10	0.16	0.53
Toilet to main door	0.79	0.79	0.21	0.25	0.40
Complete trajectory	0.98	0.98	0.03	0.13	0.78

**Table 9:** Comparison with related works.

Paper	SLAM Landmark	Testbed Size (Approx.)	Precision
[14]	Corners of the indoor environment	20 m × 20 m	1.34 m
[15]	RFID tags	30 m × 40 m	2 m
[16]	WIFI routers	60 m × 10 m	2.22 m ± 1.25 m
[17]	Human activities	10 m × 10 m	1.16 m ± 0.07 m
[18]	BLE beacons	90 m × 37 m	3.25, 4.69 (using 24 and 48 beacons)
[19]	BLE beacons	16 m × 7 m	1.05 m ± 0.44 m
This study	BLE beacons	20 m × 10 m	1.02 m in older adults 0.98 m in adults

## Data Availability

Dataset and source code available on https://github.com/jesusceron/SLAM_HAR_IL (accessed on 10 March 2022).

## Appendix A

Figure A1, Figure A2, Figure A3 and Figure A4 show the location tracking performed for the participants in the older adults and control groups.
